# 

**Published:** 1893-12-16

**Authors:** 


### CONTENTS.

**Free or Sober?** - 161
**Around the Hospitals** - 162
**On Oxygen Administration.** By Sir Benjamin Ward Richardson, M.D., F.R.S. - 163
**Democracy and the Hospitals** - 164
**Modern Aspects of Inflammation** - 165
**The Hospital Clinic**—
Disease of Dietetic Origin. By John Beddoe, B.D., M.D., F.R.S., Late Physician to Bristol Royal Infirmary - 169
Paddington Green Children's Hospital—Treatment of Hip-Joint Disease - 170
Lavage of the Intestinal Tube - 171
**Editor's Letter-Box.**—The Medical Defence Union and The London and Counties Medical Protection Society - 172
**The Field of Science** - 16
**Annotations.**—A Little More Art—The Carriage of the Dead by Railways—Beef, Butchers, and Buyers - 167
**Notes and News** - 168
**The Institutional Workshop**—Hospitals of the World—VII. Infectious Disease Hospitals in London - 173
Thirty-fifth Annual Report of the Commissioners in Lunacy for Scotland - 174
Practical Departments—Steam Disinfector - 174
Hospitals in India—I. The Bengal Presidency - 175
Construction Notes - 176
**Village Poor and London Hospitals** - 176
**Scraps and Gleanings** - 176
**The Book World of Medicine and Science** - vii

### EXTRA SUPPLEMENT.—THE NURSING MIRROR.

**News from the Nursing World.**—The Novel Competition for Nurses—Christmas Festivities—Poisons—Off to St. Helena—Night and Day—Nurseries for the R.B.N.A.—"The Usual Extras."—Isolation in a Tent—Tardy Reform—A Nurses' Home—An Illogical Deduction—Baltimore—Novel Methods—Short Items - ci

**On the Nursing of Diseases of the Stomach.**—VI. Dyspepsia (*continued*) - ciii

**Wants and Workers** - ciii

**Nursing in Labrador.** By Our Own Correspondent - ciii

**Nursing in Argentina.**—Trained Male and Female Nurses. I. civ

**The R.B.N.A. Conversazione.** By One of the Visitors - cv

**Minor Appiontments** - cv

**Life in an Infectious Hospital.** By A Nurse Who Worked There - cv

**Causerie from New England.**—Boston, Massachusetts - cvi

**Everybody's Opinion.**—Nurses and Anæsthetic - cvi

**Nursing in South Africa.**—Grahamstown - cvii

**Where to Go** - cvii

**Presentation** - cvii

**Notes and Queries** - cvii

**For Reading to the Sick.**—No More Pain - cvii

**Novelties for Nurses** - cvii

**Trials of a Doctor.** By A Country Practitioner - cviii

**The Guild of St. Luke** - cviii

**The Book World for Women and Nurses** - cix

